# Surface Hydrophobicity Strongly Influences Adsorption and Conformation of Amyloid Beta Derived Peptides

**DOI:** 10.3390/molecules29153634

**Published:** 2024-07-31

**Authors:** David L. Cheung

**Affiliations:** School of Biological and Chemical Sciences, University of Galway, University Road, H91 TK33 Galway, Ireland; david.cheung@universityofgalway.ie

**Keywords:** molecular dynamics simulation, protein conformation, intrinsically disordered proteins

## Abstract

The formation of amyloid fibrils is a common feature of many protein systems. It has implications in both health, as amyloid fibrils are implicated in over 30 degenerative diseases, and in the biological functions of proteins. Surfaces have long been known to affect the formation of fibrils but the specific effect depends on the details of both the surface and protein. Fully understanding the role of surfaces in fibrillization requires microscopic information on protein conformation on surfaces. In this paper replica exchange molecular dynamics simulation is used to investigate the model fibril forming protein, Aβ(10–40) (a 31-residue segment of the amyloid-beta protein) on surfaces of different hydrophobicity. Similar to other proteins Aβ(10–40) is found to adsorb strongly onto hydrophobic surfaces. It also adopts significantly different sets of conformations on hydrophobic and polar surfaces, as well as in bulk solution. On hydrophobic surfaces, it adopts partially helical structures, with the helices overlapping with beta-strand regions in the mature fibril. These may be helical intermediates on the fibril formation pathway, suggesting a mechanism for the enhanced fibril formation seen on hydrophobic surfaces.

## 1. Introduction

The effect of surfaces and interfaces on the formation of amyloid fibrils has been investigated for a number of years [1,2], using both experimental and theoretical methods. These have been shown to affect the fibrillization process, in a manner that is dependent on the properties of both the protein and the surface. As proteins will often adsorb onto both biological and abiotic surfaces these can play an important role in modulating the fibrillization process [3], with implications for health and potential applications of proteins in biomaterials. The aggregation of amyloidogenic proteins on biological membranes [4] can play a role in degenerative diseases, such as Type-II diabetes and Alzheimer’s disease. The interaction between synthetic surfaces and amyloidogenic proteins can be exploited in the use of nanoparticles to promote or inhibit fibril formation [5]. Some of the functional roles undertaken by amyloid fibrils [6], such as mediating adhesion onto surfaces [7,8] or structuring biofilms [9], are also dependent on their interactions with surfaces.

The role of surfaces and interfaces on fibril formation arises due to a combination of two effects [10,11,12]. Due to their amphiphilic nature, proteins can readily adsorb onto surfaces, so the local concentration of proteins is higher than in bulk solution. This increases the aggregation rate so promotes the formation of fibrils. Adsorption of proteins onto surfaces can also lead to changes in protein conformation. The effect this has on fibrillization is dependent on the particular surface and protein; for human islet amyloid polypeptide (hIAPP) hydrophobic surfaces inhibit the formation of amyloid fibrils [13], despite the higher protein concentration, which can be attributed to the adoption of alpha-helical conformations that are unfavorable to fibril formation [14].

Understanding the effect of surfaces on fibrillization then relies on knowledge of protein conformation on surfaces. A number of experimental techniques have been used to investigate protein structure on interfaces [15], including sum frequency generation spectroscopy [16] and neutron reflectivity [17]. Alongside experimental methods, molecular dynamics (MD) simulation has been used to investigate the adsorption and conformation of proteins on surfaces [18]. As amyloidogenic proteins are typically intrinsically disordered [19], they present particular problems for MD simulation even in bulk solution [20], principally the need to exhaustively sample their conformational ensemble. To address this, MD simulations of amyloidogenic proteins typically take advantage of advanced simulation approaches, such as replica exchange molecular dynamics [21] (REMD) or metadynamics [22]. Such methods have been used in a number of recent studies of the structure of amyloidogenic proteins, such as amyloid beta [23,24,25,26], hIAPP [14,27], and $\beta_{2}$-microglobulin [28], on surfaces. These have shown that at surfaces, amyloidogenic proteins can adopt significantly different structures than in bulk solution, which can be related to changes in their fibrillization behavior on surfaces. For instance, the Au111 surface promotes the formation of fibril-like conformations for both amyloid beta(1-42) [24] and hIAPP [27], while suppressing these for the Aβ(16–22) fragment [23,26]. Proteins can also adopt different conformations depending on the surface properties, such as different hydrophobicities [14] or for different crystal faces [26].

The large changes in behavior depending on protein and surface means that a general picture of how surfaces affect protein conformation and hence fibrillization is lacking. As significantly different behavior is seen depending on the protein, an investigation of a wide range of proteins, as well as different surfaces, is needed. As outlined above, simulation has been used to investigate the effect of surface properties on the conformation of some IDPs. This paper extends this work to consider the effect of surface hydrophobicity on the conformation of the model amyloidogenic protein Aβ(10–40), a 31-residue segment of amyloid beta (with the disordered N-terminus removed). As fibrillization of amyloid beta is enhanced on hydrophobic surfaces, this provides an interesting contrast to hIAPP that has been previously studied on the same surfaces using MD simulation [14]. Similar to hIAPP, Aβ(10–40) adsorbs strongly onto a hydrophobic surface and only transiently onto a polar surface. It also adopts significantly different conformations on the two different surfaces, as well as in bulk solution. In this case, however, the conformations on hydrophobic surfaces appear more amenable to fibril formation, suggesting a mechanism for the enhancement of its fibrillization on a hydrophobic surface.

## 2. Results

### 2.1. Aβ(10–40) Adsorbs Strongly onto Hydrophobic Surfaces

**Figure 1:**
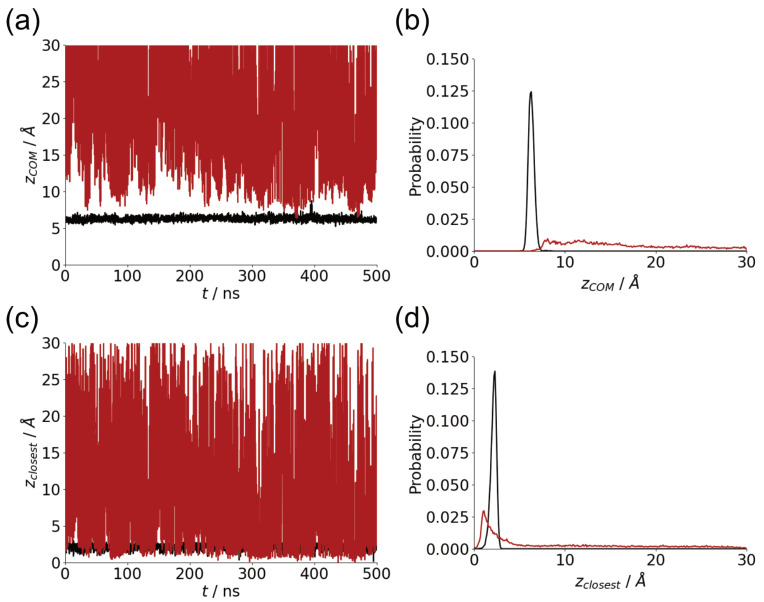
(**a**) Time variation of center-of-mass-surface separation for Aβ(10–40) on SAMch3 (black) and SAMoh (red) surfaces. (**b**) Histograms of center-of-mass-surface separation for Aβ(10–40) on SAMch3 (black) and SAMoh (red) surfaces. (**c**) Time variation of closest residue–surface separation for Aβ(10–40) on SAMch3 (black) and SAMoh (red) surfaces. (**d**) Histograms of closest residue–surface separation for Aβ(10–40) on SAMch3 (black) and SAMoh (red) surfaces.

Throughout the simulation run, Aβ(10–40) is adsorbed onto the hydrophobic surface, whereas adsorption on the polar SAMoh surface is weaker and more transient [14]. Typically the center-of-mass of the protein is ∼7 Å away from the SAMch3 surface (Figure 1a), with little variation across the simulation trajectory. For the SAMoh surface frequent desorptions from the surface are seen. Typically even when the protein is adsorbed the protein-surface separation is larger for the SAMoh surface compared to the SAMch3.

The differences in behavior can also be seen in the probability histograms of the protein-surface separation (Figure 1b); for the SAMch3 only a narrow range of separations are found, with a significantly broader distribution found for the SAMoh surface. For the SAMoh surface, the peak in this histogram is also further from the surface compared to the SAMch3 surface.

The position of the closest residue to the surface can be used as an alternative measure of the position of the protein relative to the surface (Figure 1c). For the SAMch3 surface, this behaves qualitatively similar to the centre-mass position, with a slight shift towards lower values. However, there is a larger difference between this and the center-of-mass position for the SAMoh surface, indicating protein-surface contacts even when the protein center-of-mass is far from the surface. The probability histogram of the closest residue position for the SAMoh surface shows a more defined peak compared to the center-of-mass position (Figure 1d). The peak in this is also at smaller *z* values compared to the SAMch3 surface; this closer approach arises due to the smaller size of the hydroxy group compared to the methyl group and the smaller sizes of polar side chains that are likely to be in contact with the SAMoh surface. For the SAMch3 surface the closest residue is typically a hydrophobic residue (leucine, isoleucine, valine), while smaller polar residues are typically found closest to the SAMoh surface (Table 1).

To quantify the relative adsorption strengths of Aβ(10–40) onto the different surfaces the adsorption free energy was estimated using MM-PBSA calculations (Table 2). Consistent with the strong adsorption onto the hydrophobic surface, $\Delta_{ads}G$ on the SAMch3 surfaces is significantly lower than on the SAMoh surface. This remains the case when $\Delta_{ads}G$ is calculated on only adsorbed conformations of Aβ(10–40) on the SAMoh surface. In all cases $\Delta E_{MM}$ is negative, indicating favorable protein-surface interactions, with the solvation contribution being unfavorable due to the desolvation of the polar protein surface. Similar behavior has been seen for lysozyme [29] and fibronectin fragments [30] on hydrophobic surfaces. This is particularly significant when considering the adsorbed conformations on the SAMoh surface due to the displacement of water molecules from the vicinity of the polar surface.

### 2.2. Hydrophobic Surfaces Lead to Long-Lived Contacts with Specific Protein Residues

**Figure 2:**
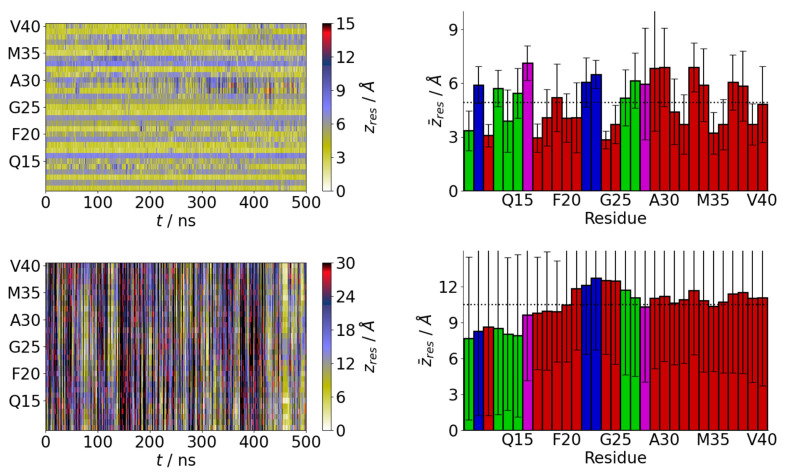
Time variation of residue–surface separations (**left**) and average residue–surface separations (**right**) for Aβ(10–40) on SAMch3 (**top**) and SAMoh (**bottom**) surfaces. Red, green, blue, and magenta denote hydrophobic, polar, negatively charged, and positively charged residues, respectively. Dotted line shows average separation for all residues.

More information on the interaction of the protein with different surfaces can be found by considering the residue positions. On the SAMch3 surface, there are long-lived contacts with the surface (Figure 2). As would be expected these are typically hydrophobic or aromatic residues. This can also be seen in the average residue–surface separations, where the closest residues are typically hydrophobic. An exception to this is the N-terminal tyrosine, which interacts with the hydrophobic surface through its side chain aromatic ring.

More variation is seen on the SAMoh surface, where contacts are typically more transient. Consistent with the center-of-mass behavior (Figure 1a), frequent desorptions from the surface are seen. While the polar N-terminus is most likely to be close to the surface, contacts with most of the protein occur. In particular, the hydrophobic C-terminus can also be found near the surface, potentially due to interactions with the charged terminus. The tendency for the N-terminus to be closer to the surface can also be seen in the average residue–surface separations. Compared to the SAMch3 surface the average separations are typically larger. The transient nature of adsorption onto the SAMoh surface is also shown by the large uncertainties in the average positions, compared to the hydrophobic surface.

**Figure 3:**
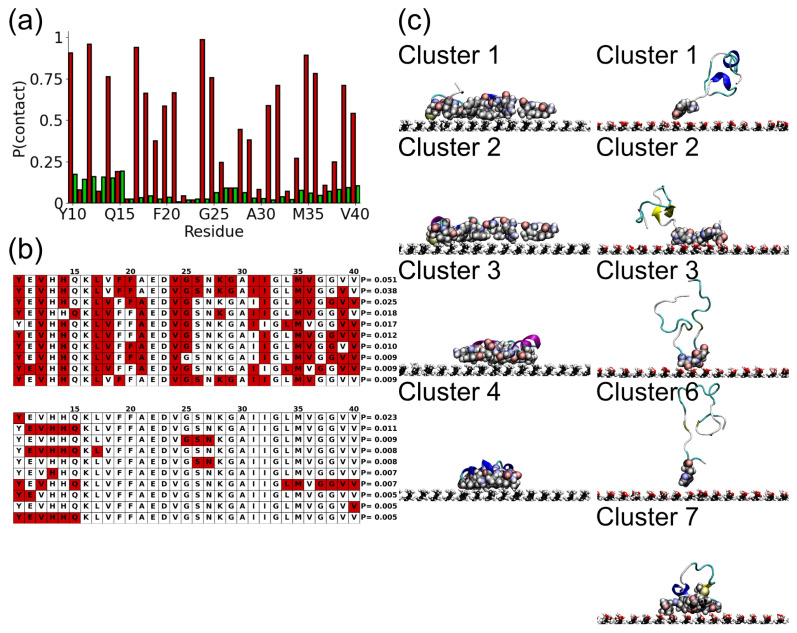
(**a**) Surface contact probabilities for Aβ(10–40) on SAMch3 (red) and SAMoh (green) surfaces. (**b**) Illustration of most likely contact clusters for Aβ(10–40) on SAMch3 (**top**) and SAMoh (**bottom**). (**c**) Snapshots of representative conformations for selected contact clusters. The left and right-hand columns show SAMch3 and SAMoh surfaces, respectively. Residues in contact with surface shown by VDW spheres.

The residues involved in surface adsorption can also be examined through the surface contact probability (Figure 3a). A residue is considered to be in contact with the surface if the closest heavy atom is within 3.5 Å (approximately the VDW diameter of a carbon atom) of the surface. For almost all residues this is higher for the SAMch3 surface, due to the stronger binding to this surface. It is particularly high for some residues (V12, L17, V24), indicating that these play a significant role in the adsorption onto the hydrophobic surface. There is a lower probability of surface contact on the SAMch3 for the charged residues, in particular, for the E22—D23 pair near the center of the protein.

On the SAMoh surface higher contact probabilities are found towards the N- and C-termini and in the short hydrophilic G25—K28 segment. The probability is highest near the N-terminus, due to the concentration of hydrophilic residues in this region.

Due to restrictions in the protein conformation, the adsorption of residues onto the surfaces is not independent and only certain combinations of residues in contact with the surface are likely. To investigate this the adsorbed conformations were clustered based on the residues in contact with the surface (Figure 3b). For the SAMch3 surface, the most likely contact clusters involved similar residues, with the V12, L17, and V24 residues being involved in all these. Only a small number of residues are not found in contact with the surface in any of the most likely clusters. While typically these are hydrophilic, the slightly hydrophobic A30 residue is not found in contact with the surface. In some of the clusters, the residues on either side of this (K28—G29 and I31—I32) are in contact with the surface, so contact between A30 and the surface would be sterically unfavorable. The similarity between the residues involved in adsorption is consistent with the strong adsorption onto the SAMch3, where the long-lived contacts between some, primarily hydrophobic, residues restrict the different conformations that can be adopted [14,26].

More variation is seen for the SAMoh surface. Notably, each cluster tends to involve fewer residues, with some involving only a single residue. Consistent with the residue–surface separations, residues near the N-terminus are commonly involved, in particular, the hydrophilic E11—Q15 segment. In most cases only a single region is involved (either near the termini or the S26—N27 region near the center); however, in one cluster (cluster 7) both the protein termini contact the surface.

It should be noted that adsorption of the termini onto the SAMoh surface is enhanced by the charge on these. The addition of neutral capping groups onto these has been shown to affect the behavior of amyloidogenic peptides on surfaces [31], so different behavior may be seen in those cases. However, as the N-terminus largely consists of polar and charged residues, adsorption of the N-terminus onto polar surfaces is still likely even when there is a neutral capping group. A greater difference may be seen for the more hydrophobic C-terminus.

The differences in the residues in contact lead to different conformations of the protein on the two surfaces, which can be seen in simulation snapshots (Figure 3c). On the SAMch3 these are similar for all the contact clusters, with the protein lying flat on the surface. A wider range of conformations is seen for the SAMoh surface. As only a small number of residues are in contact with the surface, the remainder of the protein adopts a range of conformations in solution.

**Figure 4:**
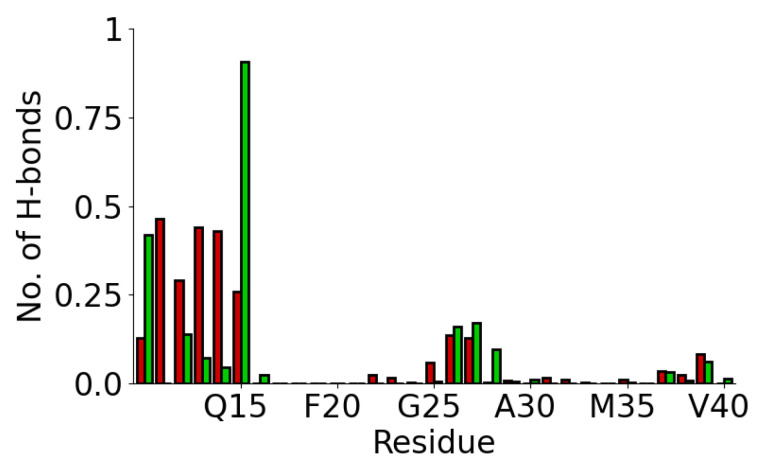
Average number of hydrogen bond acceptors (red) and donors (green) for each residue for Aβ(10–40) on SAMoh surface.

Due to the hydroxy group in the surface ligands, the SAMoh surface can form hydrogen bonds with the protein (Figure 4). The number of hydrogen bonds formed between the surface and each residue is in line with the contact probability, with residues near the termini and a small segment near the protein center forming these most commonly. In most cases, residues accept more hydrogen bonds. Exceptions to this are Y10, which has the protonated amine group in the terminus, and Q15, which has a side chain amide group.

### 2.3. Aβ(10–40) Adopts Partially Alpha-Helical Conformations on Hydrophobic Surfaces

**Figure 5:**
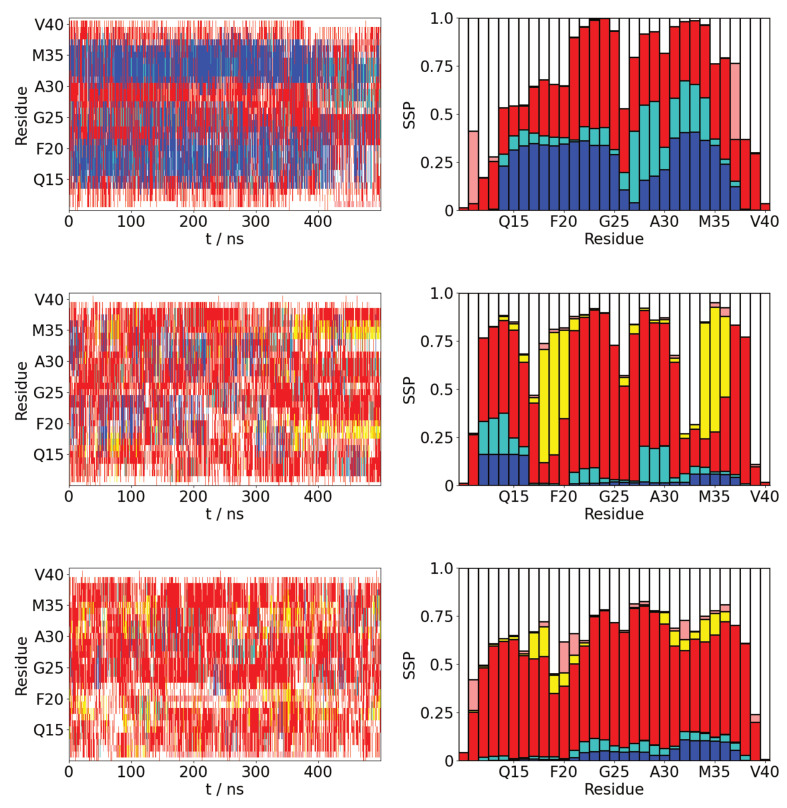
Time variation of secondary structure (**left**) and average secondary structure propensities (SSP) (**right**) for Aβ(10–40) for (**top** to **bottom**) SAMch3, SAMoh, and bulk solution. α-helix, β-strand, turn, 3/10-helix, beta-bridge, and random coil denoted by blue, yellow, red, cyan, pink, and white respectively.

In common with other IDPs [14,26], the strong interaction between the hydrophobic surface and Aβ(10–40) leads to a large difference in its conformation, compared to polar surfaces and bulk solution. Notably, this induces the formation of two alpha-helical regions, containing residues Q15—A21 and I31—V36 (Figure 5), that are stable across the whole of the simulations. These are similar to helical regions found for Aβ(10–40) on lipid bilayers [32], where the penetration of the protein into the head groups exposes it to a similar hydrophobic surface. They are also similar to those seen in a previous study of Aβ(10–40) on the air-water interface [33]. These regions also contain the beta-strand regions found in fibrils [34], suggesting that this structure may be similar to a helical intermediate state on the fibrillization pathway. Such helical structures have been observed experimentally for amyloidogenic proteins on hydrophobic-hydrophilic interfaces, such as the air-water interface [35] and lipid bilayers [36].

**Figure 6:**
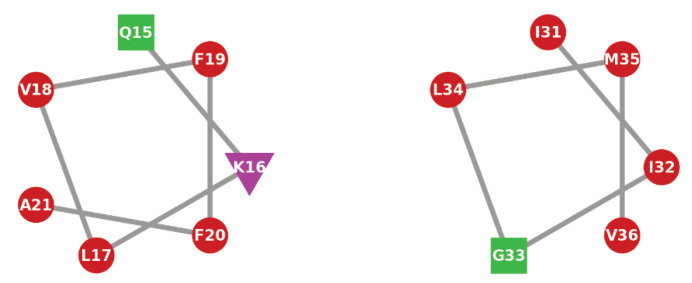
Helical wheel projections for helices formed by residues 15–21 (**left**) and 31–36 (**right**) for Aβ(10–40) on SAMch3 surface.

A common driving force of the formation of helices is the partitioning of hydrophobic sidechains into hydrophobic environments, forming amphipathic helices. To examine whether this is the case here, helical wheel projections for the two alpha-helical regions were generated (Figure 6). For the Q15—A21 helix, there is a hydrophobic face, consisting of residues L17, V18, F20, and A21, with the hydrophilic Q15 residue on the opposite face. The second helix (I31—V36) consists largely of hydrophobic residues. From the contact clusters (Figure 3b), the isoleucine, methionine, and valine residues in this region, which form one face of the helix, are typically in contact with the surface.

For the SAMoh surface and bulk solution Aβ(10–40) adopts a similar secondary structure. It is dominated by turn and random coil with a much smaller tendency for helix formation than on the SAMch3 surface. There are two small regions (V18—F20 and L34—V35) that have a tendency to form beta-strands—the probability of forming beta-strands is slightly higher on the SAMoh surface compared to bulk solution. Notably, these are not typically found in contact with the surface (Figure 3), suggesting the surface does not play a direct role in their formation. There is also a small region near the N-terminus (E11—K16) that shows a slight tendency for helix formation on the SAMoh surface. This region is relatively hydrophilic (containing five polar or charged residues) so this helix formation may facilitate favorable interactions with the polar SAMoh surface.

The differences in the secondary structure are reflected in the number of backbone hydrogen bonds (Table 3). For the SAMch3 the average number of alpha-helical and 3–10 hydrogen bonds is higher than in bulk solution and on the SAMoh surface. The dihedral offset function, which characterizes the beta-strand character of the protein, is lower on the SAMch3.

While large differences are seen in the secondary structure, changes in the tertiary structure are smaller in the different environments (Table 3). On the SAMch3 surface, the radius of gyration and the largest two eigenvalues of the gyration tensor are slightly larger than on the SAMoh surface and in solution, suggesting that the structure is more linear on the hydrophobic surface. The differences are, however, typically within one standard deviation of each other. $G_{min}$ is smaller on the SAMch3 surface—this is consistent with the protein lying flat on the surface (Figure 3c).

### 2.4. Changes to Surface Chemistry Lead to Qualitatively Different Conformational Ensembles for Aβ(10–40)

As it is intrinsically disordered Aβ(10–40) exists in an ensemble of different conformations. Previous studies have shown that this ensemble changes on surfaces [14,23,24,26], affecting both the number of conformations and the conformations that the protein can adopt. Using a cluster analysis the number of conformations found for each simulation has been determined (Table 4). The cluster analysis shows that the size of the conformational ensemble is different on the two surfaces and in bulk solution. $N_{conf}$ is lowest for the SAMch3 surface, with the reduction in the number of conformations being caused by the strong interaction between hydrophobic residues and the surface, which reduces the ability of the protein to adopt different conformations. The number of conformations on the SAMoh surface is lower than in bulk solution. These changes are also reflected in the conformational entropy, which increases with $N_{conf}$. The changes in the number of conformations and the conformational entropy are consistent with previous studies of IDPs on surfaces of different hydrophobicity [14].

**Figure 7:**
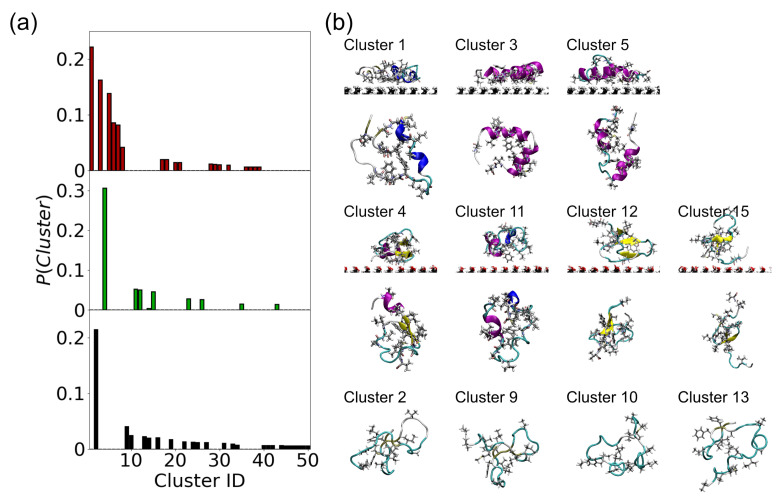
(**a**) Probability of highest ranked clusters for Aβ(10–40) (**top** to **bottom**) SAMch3, SAMoh, and bulk solution. (**b**) Snapshots of selected conformations found from the combined cluster analysis. Top, middle, and bottom show most probable clusters on SAMch3 and SAMoh surfaces and from bulk solution. For surface simulations top and bottom show side-on and top-down views, respectively.

To examine the overlap between the conformational ensembles a cluster analysis was performed on the combined trajectories for all three systems [26] (for the SAMoh surface only adsorbed conformations were considered). Considering the probability of each cluster showed that there is little overlap between the conformations that Aβ(10–40) adopts for the different systems (Figure 7a), suggesting that the conformational ensembles are significantly different from each other. In particular, the most likely conformations are different in each case, suggesting that Aβ(10–40) adopts different conformations on the two surfaces and in bulk solution.

The conformations are qualitatively different from each other. On the SAMch3 surface, helical conformations are found, reflecting the typical secondary structure found (Figure 5). These conformations also lie flat on the surface, consistent with the residue–surface separations. For the SAMoh surface and in bulk solution, more variation in the structures is found. In particular, the SAMoh surface induces a mixture of different secondary structure motifs, while in bulk solution disordered conformations are typically formed.

## 3. Model and Methodology

### 3.1. System Model and Construction

The simulated systems consist of a single Aβ(10–40) protein molecule on either a self-assembled monolayer or in bulk solution. The initial structure of Aβ(10–40) was taken from the experimental NMR structure (1IYT [37]), with the first nine and last two residues removed. Acidic and basic residues, along with the termini, were charged, as appropriate for pH = 7. The surfaces consisted of an an alkylthiol self-assembled monolayer containing 320 (20 × 16) chains, arranged in the $\sqrt{3}\times \sqrt{3}$ R3 geometry. Two different surfaces were considered, containing either hydrophobic (R = CH_3_) or hydrophilic (R = OH) ligands (denoted as SAMch3 and SAMoh). To mimic the effect of strong anchoring onto an underlying surface the positions of the terminal sulfur and hydrogen atoms of the chains were fixed throughout the simulation.

All systems were constructed using the Gromacs pdb2gmx, solvate, and genion [38,39,40] utilities (versions 4.6.7). For the surface simulations, the protein was initially placed 20 Å from the surface, where the surface was defined as the average *z* position of the terminal heavy atoms. All systems were solvated and counter-ions were added to neutralize the protein. The systems were initially energy minimized using the steepest descent algorithm followed by short (20 ps) NVT simulations (at 300 K), first with the positions of the heavy atoms in the protein restrained to their initial positions by harmonic potentials (with force constant 2.4 kcal mol^−1^ Å^−2^), then without the position restraints. For the bulk solution a short (20 ps) NpT-simulation was then performed.

The system was modeled using the Charmm22* [41,42,43] force field, charmm general force field [44], and charmm-TIP3P [45,46] for the protein, surface ligands, and water, respectively.

### 3.2. Simulation Method

**Figure A1:**
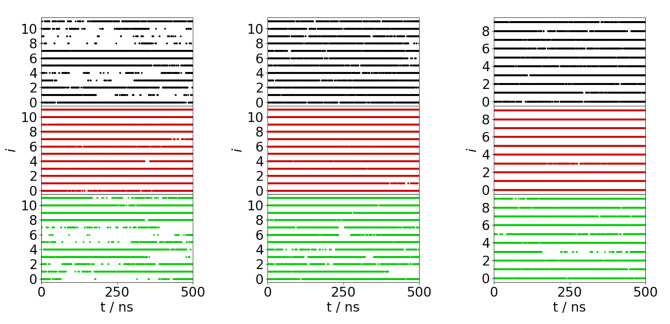
Plot of scaling factor (*i* denotes $\beta_{i}$) for Aβ(10–40) on SAMch3 surface (**left**), on SAMoh surface (**centre**), and in bulk solution (**right**). Graphs show (from top to bottom) replicas with i=0, 6, and 11 for surface simulations and i=0, 5, and 9 for solution at *t* = 0.

Replica exchange with solute tempering (REST) [47,48] was used to enhance the sampling of protein conformations. Compared to normal replica exchange molecular dynamics [49] the temperature varies for only one part of the system, in this case the protein. This reduces the number of replicas needed to span a given temperature range. The temperature scaling was performed by scaling the protein–protein and protein–solvent interactions by a factor depending on the temperature of each replica, according to [48]

(1) $$E_{i}=\beta_{i}E_{pp}+\beta_{i}^{1/2}E_{ps}+E_{ss}$$

where $E_{pp}$, $E_{ps}$, and $E_{ss}$ are the protein–protein, protein–solvent, and solvent–solvent interactions, respectively, and $\beta_{i}=T_{0}/T_{i}$ is the scaling factor. For all systems, the temperature was in the range of 300 K to 440 K; for the surface simulations 12 replicas were used, while 10 replicas were used for the solution simulations. The scaling factors and temperature for the different replicas are given in Table 5. Exchange attempts between neighboring replicas were attempted every 500 time steps (1 ps). Transitions between different temperatures and acceptance rates are given in Figure A1 and Table A1.

**Figure A2:**
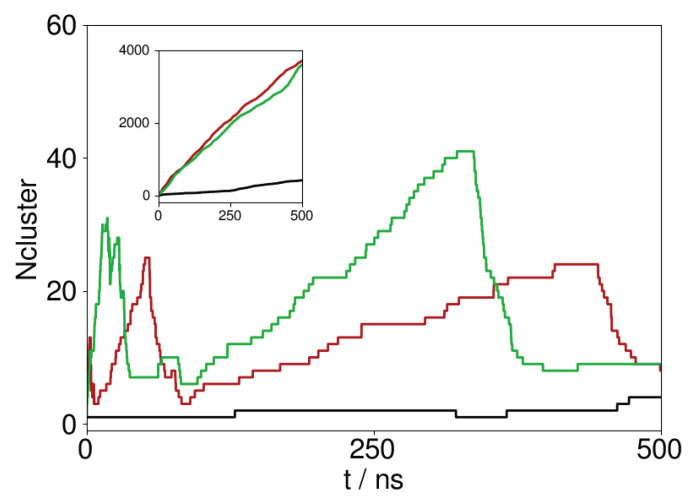
Variation in number of clusters found with simulation time. Main figure shows clusters with free energy within 2 kcal mol^−1^ of the ground state, inset shows total number of clusters. Black, red, and green denote SAMch3, SAMoh and solution simulations, respectively.

For all the simulations the temperature was set to 300 K using a velocity rescaling algorithm [50], with a relaxation time of 0.2 ps. The pressure in the bulk simulations was set to 1 atm using the Parrinello–Rahman barostat [51] (relaxation time 2 ps). For the surface simulations, the system was periodic in the *x* and *y* directions with the system contained in the *z*-direction walls using walls interacting through an integrated 9-3 LJ potential. The bulk simulations were periodic in all directions. A cutoff of 12 Å was used for the van der Waals and short-range electrostatic interactions. Long-range electrostatic interactions were evaluated using a Particle Mesh Ewald [52] sum with a Fourier spacing of 0.16 nm. Reciprocal space grids of 36 × 36 × 36 (bulk solution) and 40 × 40 × 160 (surface) were used. A time step of 2 fs was used, with the LINCS algorithm [53] used to constrain bonds containing hydrogen atoms. Simulations were performed using the Gromacs simulation package (version 4.6.7) [38,39,40], using the REST simulations implemented using the PLUMED library [54] (version 2). Simulation lengths were 500 ns, consisting of 400 ns of equilibration and 100 ns of production. Equilibration was judged by considering the number of unique conformations found from cluster analysis (Figure A2), with equilibration being achieved once the number of low energy clusters (with free energy within 2 kcal mol^−1^ of the most populated cluster) had reached a maximum.

### 3.3. Simulation Analysis

To analyze the simulations a combination of Gromacs utilities [38,39,40] and in-house Python scripts (using the MDAnalysis library [55] (version 2.4.3)) were used. The secondary structure analysis was performed using the STRIDE algorithm [56]. To analyze the helical content of the protein the number of α-helical and 3/10-helical hydrogen bonds [57]

(2a) $$\begin{array}{cc} N_{\alpha -HB} & =\sum_{i=1}^{N_{HB}}\frac{1-{\left(r_{i}/r_{0}\right)}^{n}}{1-{\left(r_{i}/r_{0}\right)}^{m}} \end{array}$$

(2b) $$\begin{array}{cc} N_{3/10-HB} & =\sum_{i=1}^{N_{HB}}\frac{1-{\left(r_{i}/r_{0}\right)}^{n}}{1-{\left(r_{i}/r_{0}\right)}^{m}}. \end{array}$$

where $r_{0}$ = 2.5 Å, *n* = 8, *m* = 12. The sums run over all potential α-helical (Equation (2a)) and 3/10-helical (Equation (2b)) hydrogen bonds, i.e., between backbone carbonyl oxygens and amine hydrogens separated by four or three residues, respectively. The dihedral offset function

(3) $$DH=\frac{1}{2}\sum_{i=1}^{N-1}\left(1+\cos\left(\phi_{i}-\phi_{ref}\right)\right)+\left(1+\cos\left(\psi_{i}-\psi_{ref}\right)\right).$$

was used to analyze the similarity to β-strands. The sum runs over the ϕ and ψ angles of the protein residues and the reference angles are $\phi_{ref}=-2.36$ rad and $\psi_{ref}=2.36$ rad, corresponding to an ideal β-strand with alternating residues on opposite sides of the protein backbone.

The protein size was characterized by the radius of gyration

(4) $$R_{g}^{2}=\frac{1}{N}\sum_{i=1}^{N}{\left(r_{i}-r^{com}\right)}^{2}$$

where $r_{i}$ is the position of the *i*th atom and $r^{com}$ is the protein center of mass and the sum runs over atoms in the protein and the eigenvalues of the gyration tensor

(5) $$G_{\alpha \beta }^{2}=\frac{1}{N}\sum_{i=1}^{N}\left(r_{i\alpha }-r_{\alpha }^{com}\right)\left(r_{\beta }-r_{\beta }^{com}\right),\alpha ,\beta =x,y,z.$$

Simulation snapshots were generated using the VMD1.9.4 (Visual Molecular Dynamics) [58] program. A cluster analysis, using the method of Daura et al. [59], using a cut-off of 3 Å, was used to determine the unique conformations adopted by the protein. All analysis was performed for the β=1 replica (the only physically relevant replica).

### 3.4. Calculation of Adsorption-Free Energy

To quantify the adsorption strength, MM-PBSA calculations [60] were used to estimate the adsorption-free energy ($\Delta G_{ads}$). $\Delta G_{ads}$ was calculated from the difference between the free energies of the protein-surface system ($G_{protein-surf}$) and protein ($G_{protein}$) and surface ($G_{surf}$) on their own

(6) $$\Delta G_{ads}=G_{protein-surf}-G_{protein}-G_{surf}.$$

This used redthe single trajectory approach, where the free energies were calculated from a single simulation of the system.

The free energy for each system was calculated from the sum of the molecular mechanics ($E_{MM}$) and solvation ($G_{solv}$) energies

(7) $$G=E_{MM}+G_{solv}=E_{MM}+G_{PB}+G_{SA}.$$

where $E_{MM}$ is the molecular mechanics energy and $G_{solv}$ is the solvation energy, which is divided into polar solvation ($G_{PB}$) and non-polar ($G_{SA}$) contributions. As in previous work, the conformation entropy is neglected due to the inaccuracy associated with its calculation and the limited influence this has on the calculated values [61]. The molecular mechanics energy is given by the sum of the internal (bonded), VDW, and electrostatic energies

(8) $$E_{MM}=E_{int}+E_{vdw}+E_{elec}$$

For the single trajectory method the internal energy of the protein-surface complex is the same as the internal energies of the protein and surface added together. The non-polar solvation energy was calculated according to [62]

(9) $$G_{SA}=\gamma SASA$$

where SASA is the solvent-accessible surface area, calculated using a probe radius of 1.4 Å, and γ=0.005 kcal mol^−1^ Å^−2^ was the surface tension. The polar solvation energy was calculated using a Poisson–Boltzmann solver, with internal and external dielectric constants of 1 and 80. The MM-PBSA calculations were performed using the MMPBSA.py script [63], part of the Amber package (version 18).

## 4. Conclusions

Using replica exchange molecular dynamics simulations, the behavior of the model amyloidogenic protein Aβ(10–40) on surfaces of different hydrophobicity has been investigated. It has long been known that surfaces and interfaces can affect protein conformation. This is particularly significant for intrinsically disordered proteins, where surfaces can change the ensemble of structures they adopt and cause them to adopt more ordered structures that can be amendable to aggregation into ordered supramolecular structures. Previous experimental work [1,2] has shown that the surface can affect the formation of amyloid fibrils, with the specific effect (inhibiting or promoting fibril formation) depending on the protein and surface properties. Understanding what factors control this then requires knowledge of protein conformation on surfaces, which can be provided by molecular dynamics simulations.

In common with other amyloidogenic proteins [14,64], Aβ(10–40) is found to adsorb strongly onto a hydrophobic surface, driven by the hydrophobic effect, while transient adsorption is seen for a polar surface. The strong adsorption onto the hydrophobic surface led to long-lived contacts between particular residues, which limits the number of distinct conformations the protein can adopt compared to the polar surface and bulk solution. Aβ(10–40) adopts a partially helical structure on hydrophobic surfaces, similar to that seen at other interfaces [32,33], that may be intermediate states for the formation of fibrils. This differs from the case of hIAPP, where fully alpha-helical structures, less amenable to fibrillization, were found on a hydrophobic surface [14]. The difference between the structures of these two proteins may then help explain the contrasting effect of hydrophobic surfaces on the fibrillization of these proteins. Future work can extend this to consider other amyloidogenic proteins, including those from functional amyloids, and to consider how different surface chemistries affect the aggregation and assembly of amyloidogenic proteins on surfaces [65].

## Figures and Tables

**Table 1:** Closest residue for Aβ(10–40) on SAM surfaces.

SAMch3		SAMoh	
**Residue**	**Count**	**Residue**	**Count**
I32	6653	H13	1588
L17	5672	Q15	1575
A21	4708	Y10	1328
M35	4663	N27	1138
V24	4595		
V12	3327		
V39	1950		
V18	1794		
V36	1515		
I31	1477		

**Table 2:** Adsorption free energies estimated from MM-PBSA calculations. Errors estimated from the standard deviation of the mean.

Surface	$\Delta G_{\mathrm{ads}}$/kcal mol^−1^	$\Delta E_{\mathrm{MM}}$/kcal mol^−1^	$\Delta G_{\mathrm{ads}}^{\mathrm{solv}}$/kcal mol^−1^
SAMch3	−47.1 ± 0.2	−57.2 ± 0.2	10.1 ± 0.1
SAMoh	−3.6 ± 0.2	−17 ± 1	13.0 ± 0.8
SAMoh (adsorbed only)	−7.3 ± 0.3	−43 ± 2	36 ± 1

**Table 3:** Measures of protein structure. $N_{\alpha -HB}$ is the number of alpha-helix hydrogen bonds, $N_{3/10-HB}$ is the number of 3/10-helix hydrogen bonds, DH is the dihedral offset function, $R_{g}$ is the radius of gyration, and $G_{max}$, $G_{mid}$, and $G_{min}$ are the eigenvalues of the gyration tensor. Uncertainties estimated from the standard deviation.

	SAMch3	SAMoh	Solution
$N_{\alpha -HB}$	5.4 ± 3.2	2.1 ± 1.0	1.6 ± 1.2
$N_{3/10-HB}$	8.0 ± 1.6	4.7 ± 1.5	3.7 ± 1.6
DH	31.1 ± 3.1	36.8 ± 2.5	38.0 ± 3.2
$R_{g}$/Å	10.6 ± 1.0	10.3 ± 1.4	10.9 ± 2.0
$G_{max}$/Å	8.5 ± 1.3	8.3 ± 1.6	8.7 ± 2.4
$G_{mid}$/Å	5.8 ± 0.6	4.9 ± 0.6	5.3 ± 0.7
$G_{min}$/ Å	2.6 ± 0.2	3.6 ± 0.3	3.8 ± 0.5

**Table 4:** Number of conformations found from cluster analysis and conformational entropy.

System	$N_{\mathrm{conf}}$	$S_{\mathrm{conf}}$/$k_{B}$
SAMch3	263	2.97 ± 0.04
SAMoh	456	4.11 ± 0.13
Solution	1371	4.31 ± 0.42

**Table 5:** REST scaling factors and effective temperatures.

	$N_{\mathrm{replica}}$	Scaling Factors
Surface	12	1 (300 K), 0.966 (311 K), 0.932 (322 K), 0.9 (333 K), 0.867 (345 K)
		0.84 (357 K), 0.811 (370 K), 0.784 (383 K), 0.757 (396 K), 0.731 (410 K)
		0.706 (425 K), 0.682 (440 K)
Solution	10	1 (300 K), 0.956 (313 K), 0.918 (327 K), 0.88 (341 K), 0.843 (355 K)
		0.808 (371 K), 0.775 (387 K), 0.742 (404 K), 0.711 (422 K), 0.682 (440 K)

## Data Availability

The original contributions presented in the study are included in the article, further inquiries can be directed to the corresponding author.

## Appendix A. Simulation Sampling and Convergence

To ensure that the REST simulations effectively sample the different replicas the variation of the REST scaling parameter ($\beta_{i}$) for different replicas has been monitored (Figure A1). As can be seen the replicas explore different values of $\beta_{i}$, suggesting effective sampling of the different replicas. This is also shown by the acceptance rates for swaps between neighbouring pairs of replicas (Table A1), which are above 30% in all cases.

**Table A1:** Acceptance rates for REST simulations.

	0↔1	1↔2	2↔3	3↔4	4↔5	5↔6	6↔7	7↔8	8↔9	9↔10	10↔11
SAMch3	0.447	0.427	0.424	0.407	0.412	0.422	0.439	0443	0.440	0.472	0.432
SAMoh	0.430	0.421	0.423	0.411	0.427	0.442	0.458	0.451	0.463	0.4096	0.433
Solution	0.353	0.367	0.395	0.316	0.403	0.398	0.377	0.414	0.421		

The time evolution of the number of unique conformations found (from a cluster anaylsis) is shown in Figure A2. As can be seen while the total number of conformations found increases across the simulation, the number of low energy clusters (those with energy within 2 kcal mol^−1^ of the lowest energy cluster) reaches a maximum within the simulation time and for the SAMch3 simulation is largely constant, suggests that sufficient sampling of the conformational ensemble has occurred.
